# Timing of fracture fixation for femur and pelvis fractures in patients with severe traumatic brain injury - an analysis of the TraumaRegister DGU^®^

**DOI:** 10.1007/s00068-026-03253-x

**Published:** 2026-06-22

**Authors:** Tobias Peter Bayer, Michel P. J. Teuben, Sascha Halvachizadeh, Alba Shehu, Rolf Lefering, Roman Pfeifer, Hans-Christoph Pape, Kai Oliver Jensen

**Affiliations:** 1 Department of Traumatology, University Hospital Zürich, Zürich, Switzerland; 2 Department of Orthopaedics and Traumatology, University Hospital Basel, Basel, Switzerland; 3 Institute for Research in Operative Medicine (IFOM), University Witten/Herdecke, Cologne, Germany

**Keywords:** Traumatic brain injury, Polytrauma, Femur fracture, Pelvic fracture, Damage control orthopedics, Early total care

## Abstract

**Purpose:**

Fracture fixation timing and strategy in polytrauma patients with traumatic brain injury (TBI) remain controversial. This study investigates treatment patterns and outcomes for femoral and/or pelvic fractures stratified by TBI severity.

**Methods:**

Patients in the TraumaRegister DGU® (2016–2022) with pelvic and/or femoral fractures (AIS ≥3) and TBI (head AIS ≥3) were included. Strategies were non-operative management (NOM), early total care (ETC), and damage-control orthopedics (DCO). Outcomes included treatment allocation, fixation timing, and in-hospital mortality.

**Results:**

985 patients were included (mean age 52.5, SD 26.3 years; ISS 27.8, SD 8.1). Allocation was NOM in 320 (32.5%), ETC in 336 (34.1%), and DCO in 329 (33.4%) patients. Head AIS was 3 in 48.5%, 4 in 31.1%, and 5 in 20.3%. NOM patients were older, had the highest ISS and estimated mortality, and showed the largest proportion of critical TBI (AIS 5: NOM 30.9%, ETC 14.3%, DCO 16.1%). Femoral ETC was mainly performed within the first day (median 0, IQR 0-1 days), whereas pelvic ETC was delayed with increasing TBI severity (median 3, IQR 0–5 days for head AIS 3; 5, IQR 0–7 days for AIS 4). Observed mortality was 37.2% after NOM, 9.2% after ETC, and 10.3% after DCO.

**Conclusion:**

ETC in patients with moderate TBI (AIS 3) was associated with reduced observed mortality relative to NOM and matching DCO. Increasing TBI severity shifted practice patterns to DCO/NOM. These findings suggest that critical head injuries may prolong time to definitive fixation being associated with higher morbidity and mortality.

## Introduction

Traumatic brain injury (TBI) is a leading cause of death and disability worldwide [1–4]. Up to 60% of patients with moderate to severe TBI have significant concomitant injuries, which contribute to an impaired outcome after TBI [3–6]. Managing both the TBI and concomitant injuries requires careful coordination, especially when it comes to clearing TBI patients for fracture fixation surgery [7, 8]. Over the last decades complication rates in patients with multiple injuries have decreased [8, 9]. The implementation of staged fracture care for severely injured patients has been observed to play an essential role in the improvement of post-traumatic outcomes [7, 10].

Severe TBI is associated with a high risk of neurological deterioration and other complications such as increased intracranial pressure (ICP) and hypoxia [3, 4]. Given these concerns, clinicians are faced with the dilemma of whether to prioritize neurosurgical interventions or to address major fractures, which may require early fixation to improve functional outcomes [11, 12]. Despite the well-known benefits of early fracture fixation, fracture surgery is often avoided in individuals with severe TBI due to the potential for exacerbation of ICP, monitoring difficulties, and cardiopulmonary alterations [13–15]. Fracture fixation can function as a “second hit”, amplifying the systemic inflammatory response with a surge of pro-inflammatory cytokines. This increases the risk of rising ICP, pulmonary dysfunction/ARDS and subsequent organ failure in vulnerable polytrauma patients [16].

However, the available data regarding the precise timing of definitive fixation of major fractures in the context of concomitant severe TBI remains limited. Consequently, a retrospective descriptive analysis of the TraumaRegister DGU^®^ (TR-DGU) of the German Trauma Society was conducted to examine practice patterns in the management of pelvic and femoral fractures in polytrauma patients with substantial concomitant head injury (Abbreviated Injury Scale (AIS) head ≥ 3). This analysis focused on the timing of fracture fixation and the allocation of patients to treatment strategies (NOM, ETC, DCO) for pelvic and femoral fractures, as well as the observed short-term outcomes.

## Material and methods

### Data collection

This study was designed as a retrospective descriptive registry-based analysis of treatment allocation, timing of fracture fixation, transfusion rate, and in-hospital mortality according to TBI severity and treatment strategy. A dataset extracted from the TR-DGU database was used. The study adhered to the TR-DGU publication guidelines and was registered as TR-DGU project ID 2023-024. The manuscript was approved in accordance with the TR-DGU publication guidelines. As a retrospective registry analysis of routinely collected, de-personalized data, ethical approval was not required in accordance with the regulations of the regional medical ethics associations.

The TR-DGU of the German Trauma Society (Deutsche Gesellschaft für Unfallchirurgie, DGU) was founded in 1993. The aim of this multi-center database is a pseudonymized and standardized documentation of severely injured patients. Data are collected prospectively in four consecutive time phases from the site of the accident until discharge from the hospital: (A) Pre-hospital phase, (B) Emergency room and initial surgery, (C) Intensive care unit, and (D) Discharge. The documentation includes detailed information on demographics, injury pattern, comorbidities, pre- and in-hospital management, course on intensive care unit, relevant laboratory findings including data on transfusion and outcome of each individual patient. The inclusion criterion is admission to hospital via emergency room with subsequent ICU/IMC care or admission to the hospital with vital signs followed by death before ICU admission. The infrastructure for documentation, data management, and data analysis is provided by AUC–Academy for Trauma Surgery (AUC-Akademie der Unfallchirurgie GmbH), a company affiliated to the German Trauma Society. The scientific leadership is provided by the Committee on Emergency Medicine, Intensive Care and Trauma Management (Sektion NIS) of the German Trauma Society. The participating hospitals submit their data pseudonymized into a central database via a web-based application. Scientific data analysis is approved according to a peer review procedure laid down in the publication guideline of TR-DGU. The participating hospitals are primarily located in Germany (90%), but a rising number of hospitals of other countries contribute data as well (at the moment from Austria, Belgium, China, Finland, Luxembourg, Slovenia, Switzerland, The Netherlands, and the United Arab Emirates). Currently, more than 30,000 cases from approximately 700 hospitals are entered into the database per year. Participation in TR-DGU is voluntary. For hospitals associated with TraumaNetzwerk DGU^®^, however, the entry of at least a basic data set is obligatory for reasons of quality assurance.

### Inclusion and exclusion criteria

The study included patients admitted to participating level I and II trauma centers in Germany, Austria, or Switzerland between January 2016 and December 2022. This specific study period was chosen as relevant parameters were added to the database in 2016, thereby providing a complete dataset for each included patient.

Patients with pelvic and/or femoral injuries with AIS ≥ 3 and concomitant head injury with AIS ≥ 3 were selected [17]. Patients with a concomitant injury to the thorax or abdomen with an AIS ≥ 3 were excluded from the statistical evaluation. This exclusion criterion was applied to create a more homogeneous cohort in which TBI represented the dominant relevant concomitant injury influencing fracture management. Major thoracic or abdominal injuries are independent drivers of hemodynamic instability, transfusion requirements, surgical prioritization, timing of fracture fixation, and mortality. Therefore, they would introduce competing indications for DCO or NOM. The registry dataset does not provide sufficient real-time physiological information for statistical adjustment to reliably account for these competing clinical determinants, and residual confounding would likely remain substantial.

Patients were excluded if they died in the trauma bay, were transferred out within 48 h, transferred in from another hospital, or did not receive ICU treatment.

The specific type of operative fracture fixation, including implant choice and surgical technique, is not recorded in sufficient detail in the TR-DGU. Therefore, treatment strategies were operationally defined based on the number and sequence of surgical procedures performed for the respective anatomical region. This approach was chosen to distinguish direct definitive fixation from staged fracture care within the limitations of the registry dataset.

The patients were divided into three different groups based on the surgical strategy for the observed femur and pelvis fractures. The groups were categorized as follows:

NOM: Nonoperative management.

ETC: Early total care, reflecting direct definitive fracture fixation performed in a single operative procedure per anatomical region. No general time cut-off was applied for the definition of ETC in this registry-based analysis. The actual timing of operative fixation was analyzed and reported descriptively.

DCO: Damage control orthopedics, which refers to staged fracture care. Patients with concurrent femur and pelvis fractures were assigned to the DCO group if DCO was chosen for at least one of the entities. DCO was defined as undergoing more than one surgical procedure per anatomical region.

We gathered data on patient and trauma characteristics, as well as outcome parameters. All eligible patients were included. Missing values were excluded from the analysis of the respective parameter.

### Statistical analysis

Continuous variables are reported as mean with standard deviation (SD) or, for skewed variables, as median with interquartile range (IQR). Categorical variables are presented as counts and corresponding percentages. Baseline characteristics were compared descriptively between treatment groups. Categorical baseline variables were compared using the chi-square test. Metric and ordinal baseline variables were compared using the non-parametric Kruskal–Wallis test. These analyses were performed to characterize baseline imbalance between groups. A p-value < 0.05 was considered statistically significant. No multivariable regression modelling was performed, and outcome comparisons were therefore interpreted as exploratory and unadjusted.

All statistical analyses were conducted using SPSS statistical software (version 25, IBM Inc., Armonk, NY, USA).

## Results

**Fig. 1:**
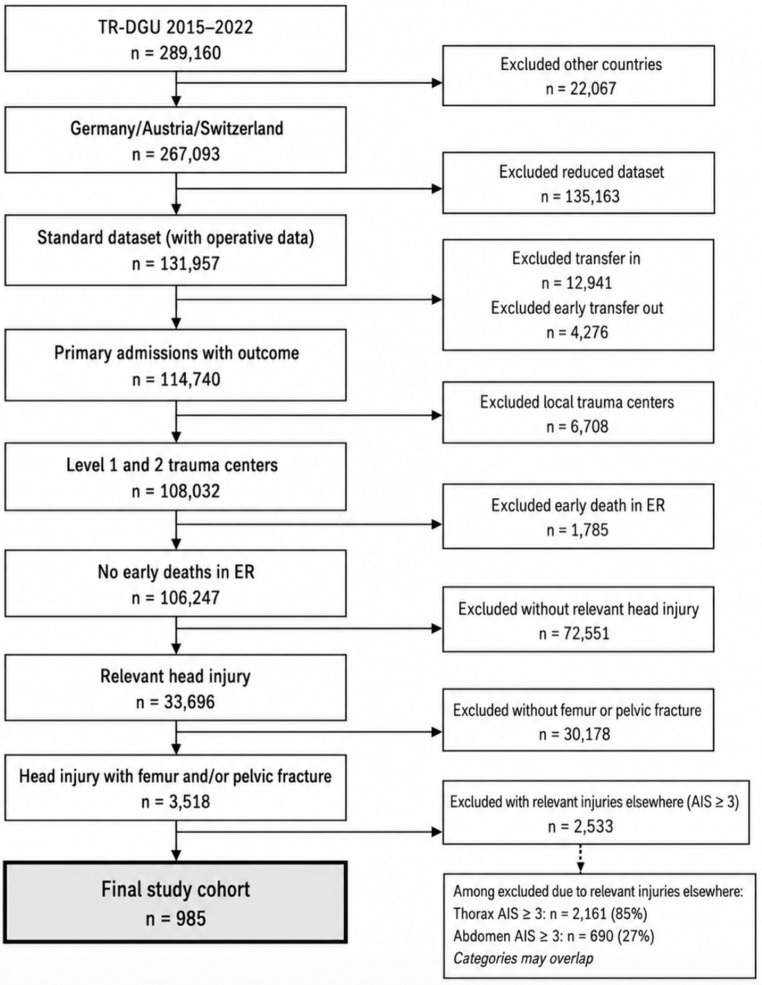
Flowchart of patient selection process from the TR-DGU

A total of 985 patients with femoral and/or pelvic fractures and concomitant TBI treated in 155 hospitals fulfilled the inclusion criteria. 289,160 cases were documented in the TR DGU between 2015 and 2022. Patients were sequentially excluded if they were treated outside Germany, Austria, or Switzerland, were included only in the reduced dataset, were transferred in or transferred out early, were treated in local trauma centers, died early in the emergency room (ER), had no relevant head injury, or had no femoral or pelvic fracture. Of 3,518 patients with relevant head injury and femoral and/or pelvic fracture, 2,533 were excluded due to additional relevant injuries in other body regions (AIS ≥ 3), including thoracic injuries in 2,161 patients and abdominal injuries in 690 patients. The patient selection process is shown in Fig. 1.

The mean age of the selected patients was 52.5 (SD 26.3) years. The mean Injury Severity Score (ISS) was 27.8 (SD 8.1) points. Basic data are shown in Table 1. 320 individuals (32.5%) were treated with NOM, 336 (34.1%) received ETC, and 329 (33.4%) received a staged DCO approach. Baseline characteristics showed relevant descriptive differences between treatment groups, with statistically significant differences observed for several variables. Patients treated with NOM were older than those treated with ETC or DCO, with a mean age of 60.5 years compared with 47.5 and 49.8 years, respectively. However, this difference did not reach statistical significance (*p* = 0.261). Injury severity was highest in the NOM group, with an ISS of 29.0 (SD 8.5), compared with 26.2 (SD 7.9) in the ETC group and 28.2 (SD 7.7) in the DCO group (*p* < 0.001). A similar pattern was observed for NISS, which was 35.5 (SD 13.4) in NOM, 30.0 (SD 9.9) in ETC, and 32.8 (SD 10.5) in DCO (*p* < 0.001). RISC-II predicted mortality was also markedly higher in the NOM group at 27.2%, compared with 11.8% in ETC and 12.6% in DCO (*p* = 0.002). Furthermore, critical TBI was more frequent in the NOM group, with head AIS 5 present in 30.9% of NOM patients compared with 14.3% in ETC and 16.1% in DCO although significance was not reached (*p* = 0.093). In contrast, ETC patients more frequently presented with head AIS 3 injuries. Blood transfusion before ICU admission was most common in the DCO group, followed by ETC and NOM (*p* = 0.052).

**Table 1:** The table summarizes the baseline characteristics of the 985 patients included in the analysis, stratified by treatment group (NOM, ETC, DCO). Described are the epidemiologic characteristics of patients for each group as well as the ISS, New Injury Severity Score (NISS) and Revised Injury Severity Classification II (RISC II) Score [18]

Variable		NOM	ETC	DCO	*p*-value
N (%)		320 (32.5)	336 (34.1)	329 (33.4)	
Males, N (%)		157 (49.1)	225 (67.0)	190 (57.8)	< 0.001
Age, mean (SD)		60.5 (26.9)	47.5 (25.7)	49.8 (25.0)	0.261
ISS mean (SD)		29.0 (8.5)	26.2 (7.9)	28.2 (7.7)	< 0.001
NISS, mean (SD)		35.5 (13.4)	30.0 (9.9)	32.8 (10.5)	< 0.001
Head injury severity	*AIS 3*,* N (%)* *AIS 4*,* N (%)* *AIS 5*,* N (%)* *AIS 6*,* N (%)*	127 (39.7)	189 (56.3)	162 (49.2)	0.093
		93 (29.1)	99 (29.5)	114 (34.7)	
		99 (30.9)	48 (14.3)	53 (16.1)	
		1 (0.3)	0	0	
Expected mortality based on RISC-II (%)		27.2	11.8	12.6	0.002
In-hospital mortality N (%)		119 (37.2)	31 (9.2)	34 (10.3)	< 0.001
Blood transfusion before ICU admission N (%)		38 (11.9)	47 (14.0)	61 (18.5)	0.052

Overall, 61.0% of the included patients were treated for a femur and 45.3% for a pelvic fracture, whereas 6.3% had both a femur fracture and a concurrent pelvic fracture. 58.4% of patients with femur fractures were treated with ETC. Only 46.4% of these had early definitive fracture fixation within the first 24 h after admission. Staged DCO was observed in 21.6% of femoral fractures. Pelvic fractures were treated with ETC in 34.8% of the cases and with staged DCO in 13.9%.

### Head injury severity and treatment strategy

**Fig. 2:**
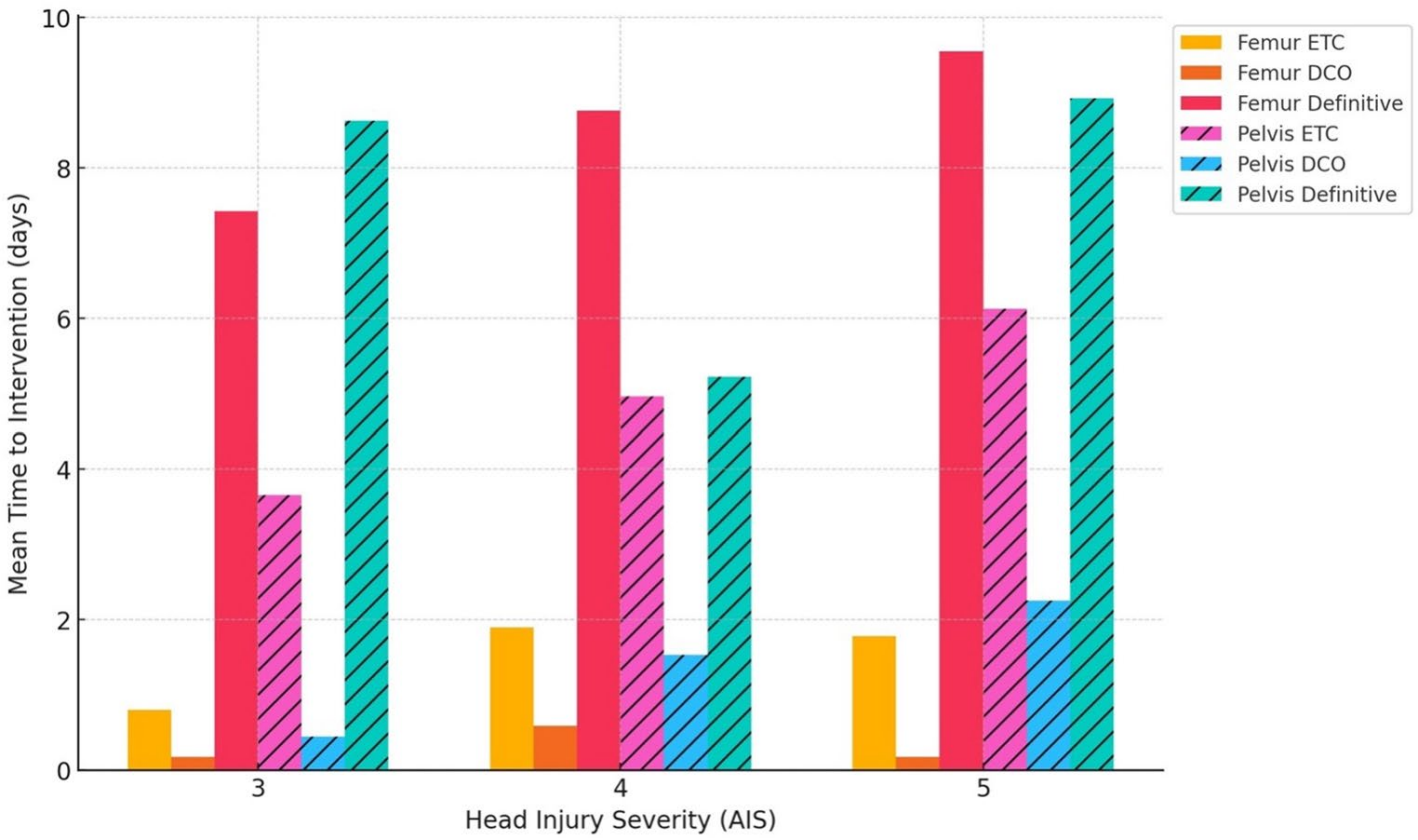
The chart displays the mean time (in days) to fracture intervention stratified by head injury severity (AIS 3, 4, 5) and fracture location as well as the modality of care showing the time to ETC, DCO, and definitive surgical care after initial DCO

Overall, 48.5% of the included patients suffered from a serious (AIS 3), 31.1% a severe (AIS 4), 20.3% a critical (AIS 5) and 0.1% (one patient) a maximal/deadly (AIS 6) head injury. In the NOM fracture fixation group 39.7% had a serious (AIS 3), 29.1% a severe (AIS 4), 30.9% a critical (AIS 5) and 0.3% a maximal (AIS 6) head injury. In the ETC group 56.3% were diagnosed with a serious (AIS 3), 29.5% a severe (AIS 4), 14.3% a critical (AIS 5) and 0% a maximal (AIS 6) head injury. In the DCO group 49.2% suffered from a serious (AIS 3), 34.7% a severe (AIS 4), 16.1% a critical (AIS 5) and 0% a maximal (AIS 6) head injury. Figure 2 shows an overview of the applied treatment strategy and timing of surgery for the different severities of head injuries.

The in-hospital mortality was 37.3% in the NOM group, 9.2% in the ETC group, and 10.3% in the DCO group. Overall, 14.8% of the patients received at least one blood transfusion in the trauma bay. The blood transfusion rates were 11.9% in the NOM, 14.0% in the ETC, and 18.5% in the DCO group.

**Fig. 3:**
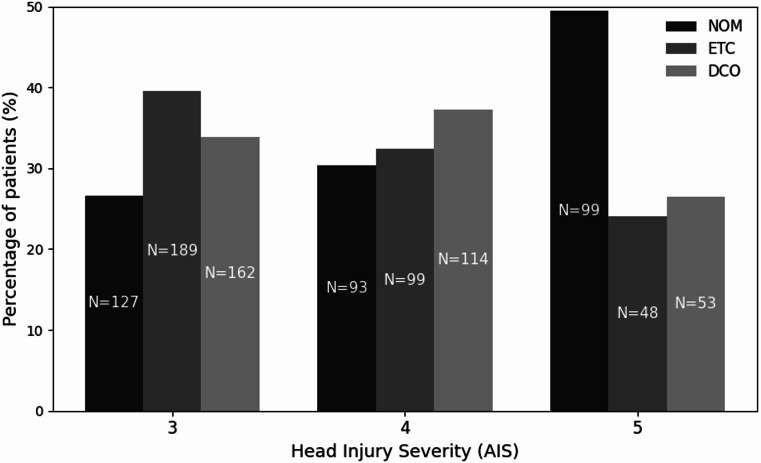
The bar chart illustrates how the choice of fracture management strategy varies with head injury severity (AIS 3, 4, 5). For each AIS grade, three adjacent bars show the percentage of patients treated with NOM, ETC or DCO

Analysis of TBI severity using the AIS revealed differences between the treatment groups. In patients with NOM 30.9% suffered critical head injuries (AIS 5), whereas only 14.3% of ETC and 16.1% of DCO patients fell into that category. Conversely, minor to moderate TBIs (AIS 3/4) were most prevalent in the ETC group (56.3%). Figure 3 displays treatment allocation according to TBI severity.

Timing of surgical intervention varied markedly according to both fracture location and TBI severity. Among femur fractures treated with ETC, fracture fixation occurred mainly within one day of admission (median 0, IQR 0–1 days).

**Fig. 4:**
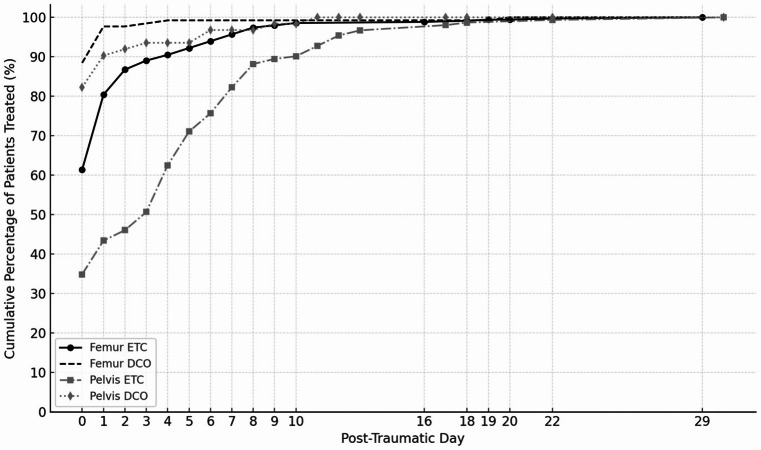
This graph illustrates the progression toward 100% first surgical treatment for each group, starting from the day of injury (Day 0) and continuing over the following days. The x-axis represents the **post-traumatic day**, while the y-axis indicates the **cumulative percentage of patients** treated with each surgical modality

For pelvic fractures ETC was performed after a median of 3 (IQR 0–5) days after admission in head-AIS 3 cases, extending to a median of 5 (IQR 0–7) days in head-AIS 4 injuries. Only a few patients with head-AIS 5injuries were treated with ETC. In the DCO cohort, initial fixation was performed within the first 48 h for 90.4% of patients. Secondary surgical intervention interpreted as definitive fracture care was performed after a median of 7 (IQR 5–10) days. Figure 4 shows the progression of the first surgical treatment up to 100% of patients treated in the different anatomical regions for the DCO and ETC treatment groups. Patients with increasing head injury severity had progressively delayed definitive fracture care, particularly for pelvic injuries. The rate of blood transfusion before ICU admission was highest in the DCO cohort (18.5%), followed by 14.0% in ETC and 11.9% in NOM patients.

## Discussion

To the best of our knowledge, the current study on patients with concurrent head and pelvic and/or femoral fractures is the first to describe that:

1. Concurrent TBI appears to be associated with the chosen treatment strategy and timepoint for fracture fixation of relevant femur and pelvic fractures. In less severe craniocerebral injuries, there is a tendency towards more frequent utilization of ETC.
2. More severe TBI was associated with increased time to surgery. More specifically, time to fracture fixation of femur or pelvis injuries, doubled in patients with head AIS 5 compared with head AIS 3 in the current dataset.

Thus, this registry study from TR-DGU indicates that the orthopedic fracture fixation strategy in polytrauma patients is markedly associated with TBI severity.

Recent evidence shows that ETC is feasible and beneficial when TBI is mild-to-moderate. A 2024 multicenter study by Zheng et al. reported that ETC for extremity fractures in patients with mild TBI did not worsen outcomes compared to delayed surgery [19]. A Delphi consensus concluded that definitive fracture fixation should be attempted within 24 h after polytrauma, provided the patient’s physiology and TBI severity permit [20]. In mild TBI cases, early fixation is recommended if there are no or no progressive intracranial trauma sequelae, an ICP of ≤ 20 mmHg, and a CPP of > 60–70 mmHg as the risk of secondary brain injury is considered lower in this subgroup [20]. In such patients, ETC for femoral and pelvic fractures can facilitate mobilization and reduce pulmonary complications without compromising neurological recovery [21]. Similarly, a systematic review by Klingebiel et al. concluded that definitive fracture fixation within 24 h appears safe and potentially beneficial in mild TBI, whereas decision-making in moderate and severe TBI remains complex and should be individualized according to neurological status, follow-up imaging, ICP/CPP parameters, and systemic physiology [22]. This concept is further supported by Kalbas et al., who emphasized that unstable physiology, ongoing intracranial priorities, and associated injury patterns remain key factors when clearing TBI patients for safe definitive surgery within 24 h [23]. In the current study, however, these physiological parameters are missing due to the registry-based design and cannot be interpreted. Tuttle et al. have demonstrated that polytrauma patients, meeting criteria for ETC, had fewer pulmonary failures and a lower rate of sepsis when ETC was initiated, whereas DCO conferred no neurologic benefit [24]. In the present dataset, observed mortality was lower in the group of patients treated with ETC (9.2%) than with NOM (37.2%) or via staged DCO (10.3%). Notably, no correction for trauma severity or matching has been conducted, as this is a descriptive study. However, the NOM and DCO groups exhibit a significantly higher average RISC-II and ISS score, which may serve as potential confounders due to selection bias. The elevated transfusion rate and mortality observed in patients with NOM and DCO likely reflect both a higher initial injury burden and the prioritization of neurosurgical stability over fracture fixation.

Additional recent literature supports a differentiated interpretation of fracture fixation timing and strategy in patients with concomitant TBI: Yu et al. reported that delaying fixation beyond 24 h in patients with mild TBI and lower-extremity long-bone fractures did not reduce complications or improve neurologic outcomes, suggesting that early fixation may be appropriate in carefully selected patients [25]. For pelvic fractures, a recent systematic review by Dormann et al. found only limited and very low-certainty evidence for early definitive pelvic stabilization, although early fixation was associated with fewer respiratory complications in the analyzed observational data [26]. These findings are consistent with our observation that femoral ETC was commonly performed early, whereas pelvic fixation was delayed with increasing TBI severity.

The present study further suggests that severe TBI may influence treatment prioritization in polytrauma. With increasing head AIS, fracture fixation was delayed, reflecting a “brain-first” approach. However, the retrospective registry study design poses limitations to causal inference. This strategy is consistent with the principle of preventing secondary brain injury at all costs [20]. Hypotension, hypoxia, or major surgical second hits can exacerbate cerebral edema and increase mortality in severe TBI. A treatment hierarchy with (1) life-saving measures, (2) CNS protection, (3) limb salvage, and (4) functional outcome is recommended [20]. This means that a polytrauma patient with elevated ICP or acute neurosurgical lesions will undergo DCO to stabilize fractures while the critical TBI is managed [27, 28]. Severe TBI has been identified as a predictor for choosing between DCO and ETC in large case series [7, 29]. Halvachizadeh et al. reported that polytrauma patients with severe TBI were more likely to receive a delayed fixation, whereas major abdominal or spinal injuries tended to trigger ETC if deemed non-lethal [29]. The data from the current study align with these findings.

In the EAST “Brain vs Bone” trial (2023), the timing of long-bone fixation did not affect 6-month neurological outcomes in TBI patients. The extent of neurological recovery primarily depended on the initial severity of TBI, rather than on whether fractures were stabilized early or late [30]. Similarly, a cohort study by Velmahos et al. revealed no increase in intracranial complications or mortality associated with ETC in patients with concomitant non-critical TBI [31]. Conversely, in severe TBI, the risks of ETC are higher: prolonged anesthesia and surgical bleeding can destabilize cerebral perfusion, and reamed intramedullary nailing releases inflammatory mediators that may worsen brain edema [20, 31]. Jaicks et al. cautioned that early femur nailing in patients with severe TBI was associated with an exacerbation of ICP and recommended delaying fixation in this subgroup [32]. Contemporary practice reflects this caution. Guidelines recommend that patients with severe TBI (Glasgow Coma Scale < 9 or intracranial AIS ≥ 4) should be managed with DCO unless their neurological and physiological parameters are stabilized [10, 27, 28].

Avoiding secondary brain damage is essential. Therefore, definitive surgery in this context is deferred until ICP is controlled, coagulation is normalized, and the patient is no longer in extremis. During that interval, priority is given to neurosurgical interventions and aggressive resuscitation to optimize cerebral perfusion. Only once the patient is both neurologically and hemodynamically stable, surgeons proceed with final fracture fixation, often under continued neuromonitoring. The observed one-week delay before definitive surgery in DCO patients is compatible with this staged treatment concept and is comparable to timelines reported in the literature [33]. In contrast to the ETC and DCO concepts, which establish specific cut-off points, more flexible trauma treatment strategies, such as Safe Definitive Surgery and Early Appropriate Care, prioritize the continued evaluation of physiological criteria to determine whether patients are suitable for fracture fixation. Instead of delaying surgery for TBI patients, these less rigid clearing protocols monitor key metrics to determine if the patient can tolerate definitive operative care [7]. Thresholds such as ICP < 15 mmHg, CPP > 70 mmHg, core temperature > 35 °C, lactate < 2 mmol/L, and controlled hemorrhage among others have been proposed as preconditions for defining a stable patient under continuous evaluation [7]. If these criteria are met, proceeding with definitive osteosynthesis may be considered. However, the TR-DGU dataset does not provide functional outcome parameters, rendering it unable to substantiate this assertion.

Determining the appropriate timing and type of fracture fixation in patients with concomitant TBI necessitates an individualized approach and interdisciplinary discussion. Secondary insults remain a risk of fracture fixation surgery in patients with severe TBI [34, 35]. Some centers employ continuous ICP monitoring and brain tissue oxygenation probes on TBI patients to detect any rise in ICP or drop in CPP in real time [34, 35]. These interventions could provide surgeons and anesthesiologists with the opportunity to intervene at early stages of cerebral compromise and potentially alter the treatment strategy to a less invasive and time-consuming alternative [34]. Despite the absence of randomized studies demonstrating the efficacy of intra-operative ICP monitoring in guiding treatment strategies, novel neuromonitoring techniques hold promise in providing more comprehensive assessments of neurological status [36].

## Limitations

Analyses based on large trauma registries, such as the TR-DGU, are invaluable for hypothesis generation but carry well-recognized methodological weaknesses. Registries depend on secondary data whose completeness and accuracy may vary. AIS miscoding rates of over 30% are reported in the literature, with complex injuries most prone to error [37]. Due to the TR-DGU enrollment criteria, which only include patients who survive hospital admission, pre-hospital and early resuscitation-bay deaths are systematically excluded. This approach results in survivorship bias, which may underestimate the true early mortality rate and can exaggerate the apparent safety of more aggressive treatment strategies [38]. Moreover, the database lacks post-discharge functional outcomes, limiting the utility for long-term effectiveness research [39].

In addition, differences in institutional protocols for TBI management, thresholds for proceeding with definitive fracture fixation, availability of neurosurgical expertise, and center-specific treatment pathways may have influenced both timing and treatment allocation, introducing systematic variation not captured in the present analysis. TR-DGU analyses have demonstrated a difference in mortality between low- and high-volume hospitals even after risk adjustment, reflecting unmeasured variation in critical resources and surgical expertise [40]. Local trauma centers were excluded from this study to minimize the potential impact of this factor. However, institutional differences are still likely to be present.

Finally, several limitations are unique to the present study. Since information on the specific operative techniques and implant choices was not available in sufficient detail, treatment allocation was inferred from the number and sequence of procedures per anatomical region. This may have resulted in misclassification of some cases, particularly when secondary procedures reflected coding inconsistencies, planned revisions, or implant exchanges rather than true staged DCO. Treatment allocation to ETC or DCO was not randomized but retrospectively analyzed. Furthermore, treatment allocation to ETC, DCO, or NOM may have been influenced by unmeasured confounders in the treating hospitals, including hemodynamic instability, shock severity, coagulation status, neurosurgical prioritization, surgeon preference, and center-specific treatment thresholds or protocols. Although patients with relevant thoracic or abdominal injuries were excluded to reduce competing indications for delayed fixation, residual confounding from physiological instability, TBI dynamics, and local practice patterns remains likely. Given the significant baseline imbalance between treatment groups, particularly regarding RISC-II predicted mortality and TBI severity, any comparisons of outcomes must be considered exploratory and unadjusted, carrying substantial risk of confounding by indication. Consequently, the present findings apply to a selected subgroup of polytrauma patients with major femoral and/or pelvic fractures and concomitant TBI, but without relevant thoracic or abdominal injuries. This limits generalizability to the broader polytrauma population. Important modifiers such as real-time coagulation status, anesthetic technique, implant choice, and exact neurosurgical timing were unavailable, preventing assessment of their influence on outcomes. Similarly, continuous physiological data such as ICP or CPP, which, as discussed, are crucial determinants for the choice of fixation, are not recorded in the registry data and thus not analyzed in this research work.

## Conclusion

In this registry-based cohort of patients with femoral and/or pelvic fractures and concomitant TBI, treatment allocation and timing of fracture fixation varied according to TBI severity. With increasing head injury severity, ETC was observed less frequently, whereas DCO or NOM were used more often and definitive fracture fixation was delayed. This suggests that decisions regarding fracture-management in routine clinical practice may be influenced by neurological injury burden and the anticipated risk of secondary brain injury. However, given the retrospective design, baseline imbalance between treatment groups, and absence of multivariable adjustment, these findings should be interpreted as observed treatment patterns rather than evidence of treatment superiority. Further prospective multicenter studies including detailed neurological, physiological, and treatment-related parameters are required to better define which patients with concomitant TBI may safely undergo early definitive fixation and which patients may benefit from staged or delayed fracture care.

## Data Availability

The data that supports the findings of this study is available from TraumaRegister DGU^®^ on reasonable request. TraumaRegister DGU^®^ restrictions apply to the availability of the data.
